# High and Low Molecular Weight Hyaluronic Acid Differentially Influences Oxylipins Synthesis in Course of Neuroinflammation

**DOI:** 10.3390/ijms20163894

**Published:** 2019-08-09

**Authors:** Dmitry V. Chistyakov, Alina A. Astakhova, Nadezda V. Azbukina, Sergei V. Goriainov, Viktor V. Chistyakov, Marina G. Sergeeva

**Affiliations:** 1 Belozersky Institute of Physico-Chemical Biology, Moscow State University, Moscow 119992, Russia; 2 SREC PFUR Peoples’ Friendship University of Russia (RUDN University), Moscow 117198, Russia; 3 Faculty of Bioengineering and Bioinformatics, Moscow Lomonosov State University, Moscow 119234 Russia

**Keywords:** hyaluronic acid, neuroinflammation, cyclooxygenase (COX-2), astrocytes, eicosanoids, oxylipins, interleukin 10 (IL-10), toll-like receptors (TLRs)

## Abstract

Hyaluronic acid (HA), a major glycosaminoglycan of the extracellular matrix, has cell signaling functions that are dependent on its molecular weight. Anti-inflammatory effects for high-molecular-weight (HMW) HA and pro-inflammatory effects for low-molecular-weight (LMW) HA effects were found for various myeloid cells, including microglia. Astrocytes are cells of ectodermal origin that play a pivotal role in brain inflammation, but the link between HA with different molecular weights and an inflammatory response in these cells is not clear. We tested the effects of LMW and HMW HA in rat primary astrocytes, stimulated with Poly:IC (PIC, TLR3 agonist) and lipopolysaccharide (LPS, TLR4 agonist). Oxylipin profiles were measured by the UPLC-MS/MS analysis and metabolites HDoHEs (from docosahexaenoic acid), -HETEs, prostaglandins (from arachidonic acid), DiHOMEs and HODEs (from linoleic acid) were detected. Both, HMW and LMW HA downregulated the cyclooxygenase-mediated polyunsaturated fatty acids metabolism, LMW also reduced lipoxygenase-mediated fatty acid metabolism. Taken together, the data show that both LMW and HMW (i) influence themselves on cytokines (TNFα, IL-6, IL-10), enzymes iNOS, COX-2, and oxylipin levels in extracellular medium of cultured astrocytes, (ii) induced cellular adaptations in long-term applications, (iii) modulate TLR4- and TLR3-signaling pathways. The effects of HMW and LMW HA are predominantly revealed in TLR4– and TLR3- mediated responses, respectively.

## 1. Introduction

The glycosaminoglycan hyaluronic acid (HA) is ubiquitously present in the extracellular matrix (ECM) of vertebrate tissues. HA exhibits diverse biological functions, including the regulation of cell adhesion, cell proliferation, the diffusion of nutrients and growth factors, and the response to tissue injury and inflammation due to its ability to interact with different receptors and other partners [1]. Recently, the biology of HA has prompted special interest due to a new view on the role of the ECM in various diseases such as osteoarthritis, fibrosis, cancer, genetic diseases and various brain pathologies [2]. The ECM of the central nervous system (CNS) comprises approximately 20% of the tissue and is presented by basal membranes, perineural networks and an intercellular matrix [3]. In the composition of the nervous system, the ECM is unique due to the absence of protein components, common to the ECM of peripheral structures. HA and proteoglycans are the major structural components [3,4,5,6] that form intricate networks and organize heterogeneous populations of neurons and glial cells into highly structured functional units of the CNS. At present, it is increasingly clear that HA does not simply provide a scaffold for cellular components of the CNS, but regulates and fine-tunes molecular processes that occur on the tissue level, playing a significant role in maintaining the homeostasis of the nervous tissue [7]. The regulation of neuroinflammation is of high importance within the broad range of HA functions.

The HA structure is rather simple with repeating disaccharide chains of N-acetyl-glucosamine and glucuronic acid that vary in length, thus low-molecular-weight (LMW HA, MW 10–500 kDa) and high-molecular-weight (HMW HA, >500 kDa) forms are distinguished [8]. Several lines of evidence indicate that HA allows for the modulation of inflammation within the CNS. A number of works has linked hyaluronan remodelling to neuroinflammation in vitro and in vivo [9,10,11]. The accumulation of HA in demyelinated multiple sclerosis lesions was shown to enhance inflammation through the activation of toll-like receptors (TLRs) 2 and 4 in immune cells, while the prevention of this accumulation alleviated injury in the autoimmune model of encephalomyelitis [12]. Furthermore, LMW HA was shown to induce astrocyte proliferation, a major step in glial reactivation [13], while HMW HA was found to suppress glial scar formation [14]. Moreover, HMW HA decreased the production of IL-1β, IL-6, TNFα and nitric oxide in microglial cells exposed to lipopolysaccharide (LPS) [15]. It is noteworthy that microglial cells are cells of immune origin, while astrocytes have an ectodermal origin and differ from myeloid cells in several major regulatory respects [16,17]. Important to note that astrocytes have widely developed TLR signaling system, including TLR2, TLR4, TLR5, TLR3, CD44 [18,19]. Taken together, these data indicate that HA might represent an important regulator of inflammatory processes which are potent enough to initiate, mitigate or fine-tune responses within the central nervous tissue; nevertheless, this issue is yet to be studied. Within the present research, we assumed that both LMW and HMW HA might initiate and/or alter astroglia-mediated inflammatory responses. To verify this assumption, we exposed mixed glial cultures, enriched in astrocytes, to LMW HA and HMW HA, with or without TLR3 and TLR4 agonists, and analysed the expression of the pro-inflammatory marker TNFα, the anti-inflammatory marker interleukin 10 (IL-10), and the release of lipid mediators, oxylipins, involved in inflammation. Oxylipins are lipid signaling molecules, produced by multi-enzymatic reactions and derived from the oxidation of polyunsaturated fatty acids [20]. Their possible role in the regulation of inflammation is shown, while yet little is known regarding their involvement in the HA-mediated cell response. Our data reveal that both LMW and HMW exogenous HA enhances the synthesis of IL-10 at the protein and at the mRNA level upon stimulation by TLR3 and TLR4 agonists. At the same time, HA does not affect the expression of pro-inflammatory cytokine TNFα in LPS and PIC stimulated cells. Further, the tested HA performs various actions of stimuli-induced TNFα expression. Therefore, the tested substances cannot be easily attributed as pro- or anti-inflammatory modulators in astrocytes. Moreover, we found that both HMW and LMW HA reduces stimuli-activated oxylipin synthesis, via the modulation of the cyclooxygenase (COX) and lipoxygenase (LOX) pathways of the polyunsaturated fatty acid (PUFA) metabolism.

## 2. Results

### 2.1. Modulation of Pro-Inflammatory Cytokine TNFα Expression by HMW and LMW HAs

**Figure 1:**
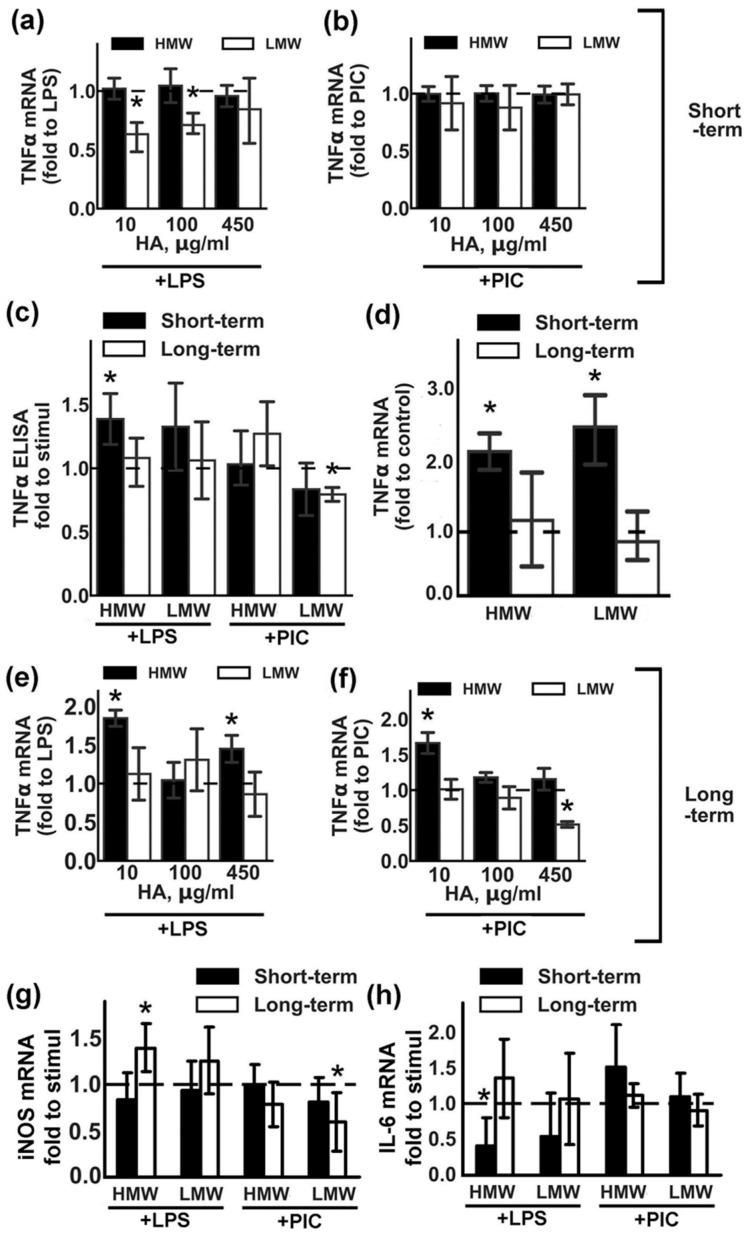
Effects of low molecular weight (LMW) and high molecular weight (HMW) hyaluronic acid (HA) on the expression of TNFα upon stimulation with TLR3 and TLR4 agonists. Astrocytes were pretreated for 0.5 h (short-term) or 48 h (long-term) with LMW (10, 100, 450 µg/mL) and HMW (10, 100, 450 µg/mL), then lipopolysaccharide (LPS) (100 ng/mL) or Poly:IC (PIC) (10 µg/mL) were added for 4 h. Relative TNFα mRNA levels were determined by real-time PCR, the data were normalized to the β-actin mRNA levels. Results in (**a**,**b**,**e**,**f**) are presented as fold-changes of TNFα relative to the LPS or PIC-stimulated cells without HA treatments. Results in (**d**) are presented as fold-changes of TNFα relative to the non-treated cells. (**c**) TNFα concentrations were measured by ELISA in supernatant from samples with long- (white bars) and short (black bars) -term HMW (450 µg/mL) and LMW (450 µg/mL) HA pretreatment followed by LPS and PIC stimulation for 4 h. Results in (**c**) are presented as fold-changes of TNFα relative to the LPS or PIC-stimulated cells. Results in (**g**,**h**) are presented as fold-changes of iNOS and IL-6 mRNA levels relative to the LPS or PIC-stimulated cells. Values represent mean ± SEM from three independent experiments performed in triplicate. * *p* < 0.05, compared with the stimulated cells (**a**,**b**,**c**,**e**,**f**), * *p* < 0.05, compared with the unstimulated cells in (**d**).

Previously, we have shown that stimulation by LPS (TLR4 agonist) or PIC (TLR3 agonist) induced TNFα expression in astrocytes [21]. Therefore, we estimated the influence of variation in concentrations and treatment time of HMW and LMW HA on LPS- and PIC-induced TNFα expression (Figure 1).

In the first set of experiments, the effects of short-term HA treatments with different concentrations were evaluated (Figure 1a,b). HA was added for 30 min, then LPS (100 ng/mL) or PIC (10 µg/mL) was added for 4 h. TNFα mRNA levels in the presence of TLR agonists without HA addition were taken as one. We found that LMW HA at concentrations of 10 and 100 µg/mL downregulated the LPS-induced mRNA of TNFα, while HMW HA had no effect on any of the tested concentrations (Figure 1a). The tested HA treatments did not modulate the PIC- induced mRNA of TNFα (Figure 1b). At the protein level, these effects of HA were only notable for LPS-induced upregulation when combined with 450 µg/mL of HMW HA (Figure 1c).

The second set of experiments allowed us to evaluate the effects of long-term (48 h) treatments with HA on LPS- or PIC-induced responses. In preliminary experiments we evaluated possible changes in the morphology of astrocytes exposed with HMW or LMW for 48 h by fluorescence microscopy and observed no visible differences between the cells (Figure S1). We also assumed the possibility that forms of HA might have modulated the expression of TNFα mRNA in non-stimulated cells under conditions involving short- and long-term expositions (Figure 1d). Short-term incubation with either LMW or HMW HA induced the two-fold TNFα expression upregulation (Figure 1d). Long-term incubation with both tested HA did not modulate TNFα mRNA levels and cells returned to initial levels of mRNA TNFα (Figure 1d). After 48 h exposition with HAs, the culture medium was changed and cells were stimulated with LPS (100 ng/mL) or PIC (10 µg/mL) for 4 h. TNFα mRNA levels in the presence of TLR agonists without HA addition were taken as one (Figure 1e,f). Long-term incubations with LMW did not influence the LPS-induced expression of TNFα mRNA (Figure 1e), but inhibited the PIC-induced upregulation of the transcript (Figure 1f), while the decrease in mRNA levels correlated with the decrease in protein levels (Figure 1c).

To assess whether the effects of LMW and HMW HA on TNFα are specific for this cytokine, we compared their effect on the expression of other inflammatory markers. iNOS (inducible nitric oxide synthase), IL-6 (interleukin 6). Cells were treated with 450 µg/mL HMW or LMW in the same experimental procedures as for the TNFα testing (Figure 1g,h). Modulation of the iNOS expression was the same as for TNFα (Figure 1g). Long-term incubations with HMW potentiate LPS-induced gene expression, while long-term incubations with LMW decrease PIC-induced gene expression. Short-term incubation with HMW decreased LPS-induced IL-6 expression (Figure 1h).

Taken together, the data indicate that we cannot attribute anti-inflammatory action to HMW HA, nor can we refer to LMW HA as a pro-inflammatory substance, as both tested variants can modulate expression levels of inflammatory markers, and these effects appear to be time- and challenge-dependent. Nevertheless, the data show that hyaluronic acids with different molecular weights modulates the astrocytes responses to the TLR agonists stimulations.

### 2.2. Modulation of Anti-Inflammatory Cytokine IL-10 Expression by HMW and LMW HA.

**Figure 2:**
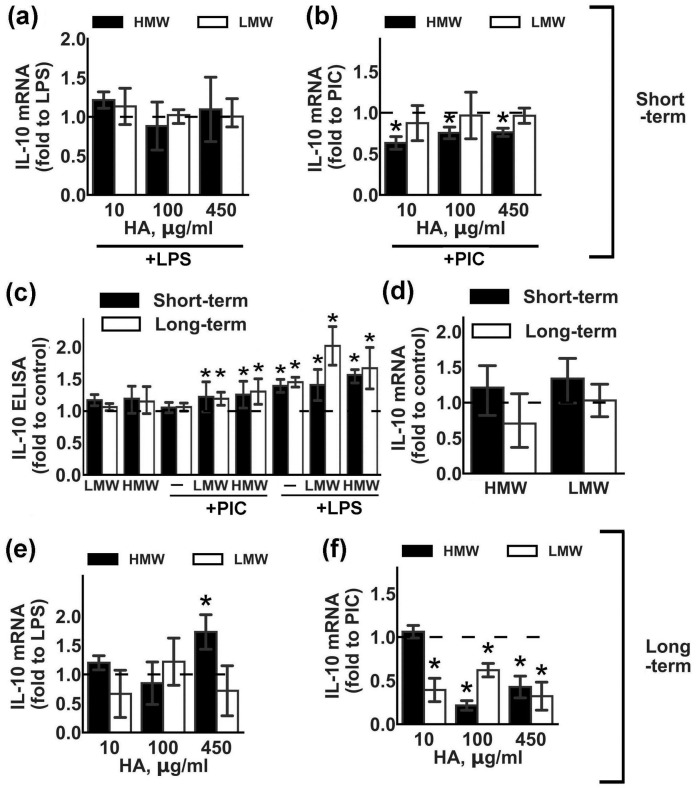
Effects of low molecular weight (LMW) and high molecular weight (HMW) hyaluronic acid (HA) on the expression of IL-10 upon stimulation with TLR3 and TLR4 agonists. Astrocytes were pretreated for 0.5 or 48 h with HMW (10,100 or 450 µg/mL) and LMW (10,100 or 450 µg/mL) HA alone or in combination with LPS (100 ng/mL) or PIC (10 µg/mL) for 4 h. Relative IL-10 mRNA levels were determined by real-time PCR, the data were normalized to β-actin mRNA levels. Results in (**a**,**b**,**e**,**f**) are presented as fold-changes of IL-10 relative to LPS or PIC-stimulated cells. Results in (d) are presented as fold-changes of IL-10 relative to the control cells. (**c**) IL-10 concentrations were measured by ELISA in supernatants from samples with long- (white bars) and short (black bars) -term HMW (450 µg/mL) and LMW (450 µg/mL) HA pretreatment followed by LPS or PIC stimulation for 4 h. Results in (**c**) are presented as fold-changes of IL-10 relative to the control cells. Values represent mean ± SEM from three independent experiments performed in triplicate. * *p* < 0.05, compared with the stimulated cells (**a**,**b**,**e**,**f**), * *p* < 0.05, compared with the unstimulated cells in (**c**,**d**).

IL-10 is an anti-inflammatory cytokine that is induced in astrocytes after stimulation with LPS or PIC [21,22]. We estimated the influence of various concentrations of HMW and LMW HA on LPS- and PIC-induced IL-10 expression (Figure 2). Short-term (Figure 2a,b) or long-term (Figure 2e,f) incubation with LMW HA did not influence LPS-induced IL-10 mRNA expression. Short-term (Figure 2b) exposition to LMW HA also had no effect, while long-term LMW exposition (Figure 2f) downregulated PIC-induced IL-10 mRNA expression for all tested concentrations. Short-term (Figure 2b) and long-term (Figure 2f) HMW exposition downregulated PIC-induced IL-10 expression, while only long-term exposition to HMW HA at a concentration of 450 µg/mL modulated mRNA IL-10 expression (Figure 2c,f). Note that, while both tested forms of HA induced TLR-mediated IL-10 release at the protein level (Figure 2c), the effects were weak but statistically significant. The addition of HAs without TLR agonists had no effect on the IL-10 release (Figure 2d). So, both tested forms of HA upregulated IL-10 protein expression in a TLR3- or TLR4-mediated manner.

### 2.3. Modulation of Oxylipin Synthesis by HMW and LMW HA.

**Figure 3:**
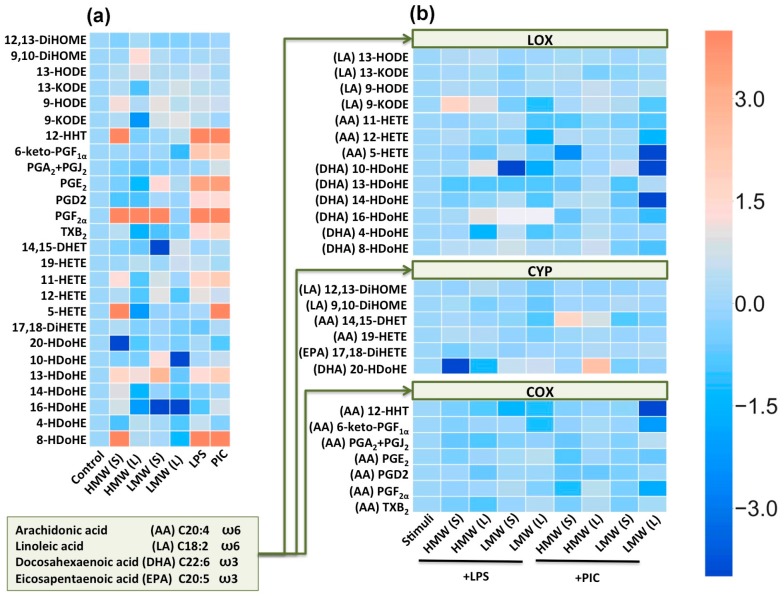
Heatmap representation of oxylipin production of n-6 and n-3 fatty acid-derived lipid mediators. (**a**) Astrocytes were treated with the high molecular weight hyaluronic acid (HMW, 450 µg/mL) and low molecular weight hyaluronic acid (LMW, 450 µg/mL) for 0.5 or 48 h, and LPS (100 ng/mL) or PIC (10µg/mL) for 4 h. Concentrations of oxylipins in supernatants were measured using UPLC-MS/MS. The heat map shows relative amounts of each lipid mediator compared to the control. The horizontal axis indicates the stimuli, while the vertical axis indicates the relative amount (ln) of each lipid mediator. (**b**) Astrocytes were pretreated for 0.5 or 48 h with HMW (HMW, 450 µg/mL) and (LMW, 450 µg/mL) HA and then stimulated with LPS (100 ng/mL) or PIC (10 µg/mL) for 4 h. The heat map shows relative amounts (ln) of each lipid mediator compared to the LPS and PIC-stimulated cells respectively. Values represent the mean from three independent experiments. Metabolites were divided into: Lipoxygenase (LOX), cyclooxygenase (COX) and cytochrome (CYP) pathways involved in their synthesis.

In addition to the release of pro- and anti-inflammatory cytokines, responses to inflammatory stimuli are characterized by oxylipin synthesis [23]. Oxylipins are formed from PUFAs via lipoxygenase (LOX), cytochrome P450 (CYP), cyclooxygenase (COX) pathways or non-enzymatically [20,23,24]. Oxylipins have tremendous effects on cellular responses, including pro- and anti- inflammatory actions via specialized plasma membrane receptors, nuclear receptors or other mechanisms [23,24]. Although many oxylipins are released in low concentrations, their effects can be summarized [25]. The recent development of mass-spectrometry allows for the analysis of oxylipin profiles. Such profiles have not been previously measured for TLR-stimulated astrocytes; therefore, we obtained oxylipin profiles for LPS and PIC stimulation (Figure 3a). Data is presented as heatmap, where the horizontal axis indicates the stimuli, while the vertical axis indicates the relative amount (ln) of each lipid mediator.

In the extracellular medium of astrocytes we obtained derivatives of arachidonic acid (AA) via LOX (11-HETE, 12-HETE, 5-HETE), CYP (14,15-DHET, 19-HETE), COX (12-HHT, 6-keto-PGF1a, PGA2, PGE2, PGD2, PGF2a, TxB2) pathways (Figure 3). We also measured derivatives of docosahexaenoic acid (DHA), produced via LOX pathways (4-, 8-, 10-, 13-, 14-, 16-HDoHE) or CYP (20-HDoHE) pathways (Figure 3). As far as we know, these are the first data concerning TLR-mediated DHA metabolism in astrocytes. Although it is known that DHA and AA are released by various isoforms of phospholipase A2 in astrocytes [26] and the LPS-induced release of DHA in astrocytes [27], the oxylipin profiles of DHA metabolites were not characterized. Surprisingly, there were also derivatives of linoleic acid (LA), via LOX (9-, 13-HODE, 9-, 13-KODE) and CYP (9,10-DiHOME, 12,13-DiHOME) pathways (Figure 3). We also measured 17,18-DiHETE, a derivative of the eicosapentaenoic acid (EPA), produced via the CYP pathway, but it was not modulated in our treatments (Figure 3a). Both LPS and PIC stimulation induced the arachidonic acid (AA) metabolism via the COX pathway (Figure 3a) and docosahexaenoic acid (DHA) metabolism via the LOX pathway (Figure 3a). The TLR-mediated synthesis of AA derivatives via the COX pathway was shown previously [20,28].

Although the oxylipin profiles for LMW HA or HMW HA actions were not estimated before, the possibility for, a link between the hyaluronan-mediated inflammation and arachidonic acid metabolism via the COX pathway was identified for monocytes and macrophages [29]; therefore, we evaluated the existence of this link in astrocytes. We compared the long-term and short-term effects of the tested HA on profiles of oxylipins. The tested treatments modulated oxylipin profiles and the effects differed for short-term and long-term applications for both LMW and HMW acids (Figure 3a). Interestingly, the effect of short-term HMW HA treatment on such metabolites as 12-HHT, PGF2α, 8-HDoHE makes it comparable to the effects of LPS or PIC (Figure 3a). Additionally the effect on 5-HETE is comparable to the PIC-treatment response. The effect of HMW HA is differing for short and long treatment, which indicates the possibility of adapting to the action of HMW. LMW HA also affects the profile of oxylipins, but is less pronounced (Figure 3a). The short-term LMW HA application induced the release of PGF2α at a level comparable with LPS or PIC stimulation, there were also notable decreases of 14, 15-DHET and 16-HDoHE (Figure 3a). Again, long-term LMW HA application reveals changing in oxylipin profiles in comparison with the short-term application (Figure 3a). In the next step, we analysed the influence of HA on LPS- and PIC-induced oxylipin profiles (Figure 3b). In Figure 3b, the amounts of stimuli-induced oxylipins were taken as one, while the alterations (compared to stimuli-induced) to every detected substance are presented as a natural logarithm (ln) (Figure 3b). We found that LMW HA modulates the LOX pathway and the effects increase upon prolonged exposition (Figure 3b), with both tested forms of HA decreasing the COX pathway activated by LPS or PIC (Figure 3b).

### 2.4. Modulation of Cyclooxygenase 2 (COX-2) Expression by HMW and LMW HA.

**Figure 4:**
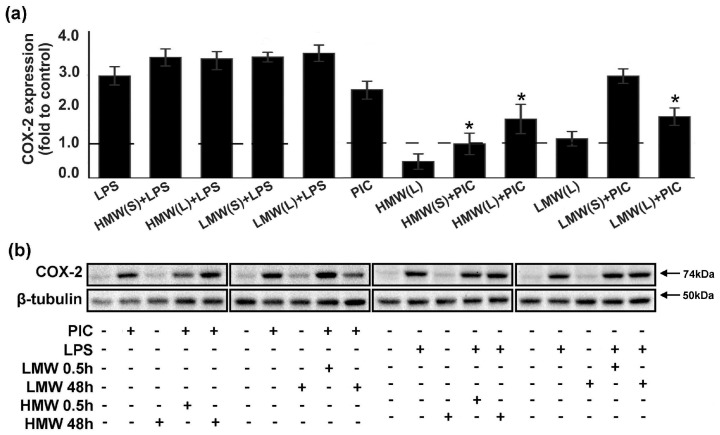
Effects of low molecular weight (LMW) and high molecular weight (HMW) hyaluronic acid (HA) on the expression of COX-2 upon stimulation with TLR3 and TLR4 agonists. (**a**,**b**) Astrocytes were pretreated for 0.5 or 48 h with HMW (10, 100 or 450 µg/mL) and (10, 100 or 450 µg/mL) alone or in combination with LPS (100 ng/mL) or PIC (10 µg/mL) for 4 h. COX-2 protein levels were evaluated by western blotting and normalized to the loading control β-tubulin. (**b**) The example is representative for three independent experiments. Values represent the mean ± SEM from three independent experiments performed in triplicate. * *p* < 0.05, compared with the stimulated cells (PIC or LPS).

Influence of HA on oxylipin profiles stimulated with PIC (Figure 3b) allows to suggest the influence of COX-2, a key enzyme of prostaglandin synthesis after inflammatory stimulation. Previously, we have shown that COX-2 is the main enzyme responsible for the LPS- and PIC-induced synthesis of appropriate substances in astrocytes [28,30]. Therefore, we analysed the modulation of LPS- and PIC-induced COX-2 expression by HMW and LMW HA under conditions of short-term and long-term exposure (Figure 4). We found that the tested forms of HA had no influence on LPS-induced COX-2 expression, while there was a significant decrease in PIC-stimulated COX-2 expression in cells exposed to LMW or HMW (Figure 4). The data concerning the COX-2 protein expression correlated with oxylipin synthesis patterns (Figure 3b). This reveals the influence of HA on TLR-mediated prostaglandin synthesis in astrocytes via the modulation of the COX-2 metabolic pathway.

## 3. Discussion

Our data shows that both LMW and HMW 1) influence alone on cytokines and oxylipins levels in extracellular medium of cultured astrocytes, 2) induced cellular adaptations for long-term applications, 3) modulate TLR4 and TLR3 signaling pathways. The effects of HMW and LMW HA are predominantly revealed in the TLR4 – and TLR3- mediated responses, respectively.

It is well known that most HA properties are size-dependent. HMW HA (> 1000 kDa) exerts anti-inflammatory effects, while LMW HA displays pro-inflammatory properties [12,31,32,33,34]. Therefore, HA itself and HA-based biomaterials have found success in a broad range of biomedical applications, connecting the treatment of inflammation and wound healing in dermatology, orthopedics, arthritis and ophthalmology [12]. In spite of the physiological significance of HAs the molecular mechanisms of their action is still not clear. Studies involving HA and TLR-mediated signaling activation are complicated because of the promiscuity of HA for several cell surface receptors, co-receptors and associated proteins. HA can bind the receptor for hyaluronan-mediated motility, TLR2, TLR4, CD44, and intercellular adhesion molecule-1 (ICAM-1), modulation of receptor complexes with TLR4-MD-2, CD44 or CD14 [1,15]. It is generally accepted that HA mediates its effects by blocking the induction of inflammatory signaling through an extracellular mechanism [1].

Within the CNS, HMW HA was reported to downregulate the proliferation of astrocytes [13,14], while the HA-dependent activation of TLR2 on immature oligodendrocytes has implications for remyelination in the case of multiple sclerosis [35]. The effect of LMW and HMW HAs on TLR-mediated cellular responses of astrocytes has not been characterized previously. Therefore, we compared their influence on two main branches of the cellular responses: Cytokines release and oxylipin synthesis. We used two protocols of HAs applications: 30 min (short-term) and 48 h (long-term) before TLR agonists stimulations for 4 h. The long-term application reveals possible involvement of feed-back mechanisms that allow cells to adapt for initial response, which can be estimated in the short-term application protocol. We found that the short-term HMW HA application induced mRNA TNFα being added along, and also potentiate LPS-mediated TNFα expression, both on mRNA and protein levels. The long-term HMW HA application also induced LPS-mediated mRNA TNFα expression. Note that 10 µg/mL and 450 µg/mL, but not 100 µg/mL have effects. Such concentration dependence in cellular responses usually reflects the multiple mechanisms of substances action. In favor of the multiple mechanisms for the realization of the effects of HA, it is also indicated that 10 µg/mL of HMW HA potentiated PIC-mediated TNFα expression. In addition, the long-term application of 450 µg/mL HMW HA induced LPS-mediated mRNA IL-10 expression, that allow to suspect the joint points of this concentration effects in LPS-mediated induction of TNFα and IL-10, possibly at the level of NF-kB, as it was shown previously [36]. HMW HA also reduced PIC-mediated IL-10 mRNA expression. In addition, HMW HA modulate oxylipin metabolism more likely not as an anti-inflammatory substance. While HMW HA are mainly affected on LPS-mediated astrocytes responses, LMW HA affected PIC-mediated responses. It decreased the agonist-mediated TNFa and IL-10 expressions after long-term incubations, decreased the COX-2 expression and reduced oxylipins metabolism. These data reveal unusual action of HAs in astrocytes on TLR-mediated signaling in comparison with other cell types. Our data point concerns the special regulatory elements in astrocytes, as well as the more complicated link between HA and TLR-mediated cellular responses. There are only a few data concerning the HA modulation of TLR3-mediated responses. It was shown that the presence of oligo-HA suppressed the PIC-induced release of IL-6 and TNFα mRNA expression in macrophages, while the TLR4 signaling pathway was involved in the manifested effects of HA [37]. Our data are in agreement with this assumption, as only the long-term exposure to LMW HA resulted in the suppression of PIC-induced TNFα expression.

In estimating the LMW and HMW HA effects on oxylipin profiles, we also revealed some special features of astrocyte sensitivity for LMW and HMW HA in comparison with macrophages and microglia. Indeed, it was previously shown that LMW HA activated the COX-2 expression and PGE2 production in human monocytes via the TLR4/MYD88 pathway [29], while HMW HA suppressed the LPS-induced COX-2 expression and PGE2 production in U937 macrophages via the down-regulation of NF-κB [38]. We found a similarity between LMW and HMW HA in astrocytes. There was no influence on LPS-induced COX-2 expression, but there was a significant decrease in PIC-stimulated COX-2 expression, which was accompanied by alterations in the oxylipin synthesis. Important to note that this is the first investigation of the HA influence oxylipin profiles and many found metabolites are still not characterized in view of the astrocytes function modulation. More or less the AA derivatives were tested in astrocytes previously and the possibility of cooperative effects of various metabolites are currently discussed [25]. Nevertheless, present data allow to conclude that both LMW and HMW modulate oxylipins metabolism via all three branches (COX, LOX, CYP). The tested HAs not only modulate TLR-mediated oxylipin synthesis but also possess their own effects when they are added alone. This conclusion is consistent with the long-term application data, which reveal changes in oxylipin profiles.

An inflammatory response is generally referred to as an immune process initiated at the level of cellular assemblies upon alterations to the homeostasis caused by the invasion of pathogens of bacterial, fungal, protozoan or viral origin, sterile injuries, or significant disbalance in metabolic profiles [39]. Previously, an inflammatory response has been attributed to cells of the immune origin, i.e., microglia cells in the brain tissue [15]. An important outcome of studies on molecular mechanisms that underlie inflammation was that an inflammatory response is not a specialized function of cells of hematopoietic origin (macrophages, neutrophils, lymphocytes etc.), but rather a fundamental attribute of all cellular types. Indeed, the inflammatory activation of astrocytes has been implicated in various CNS diseases, including multiple sclerosis, human immunodeficiency virus-associated dementia, and Alzheimer’s and Parkinson’s diseases [40]. Therefore, understanding the specificity of the inflammatory response regulation is important for developing therapeutic approaches for treating these pathologies. Our results once more allow us to emphasize the specificity of the innate immunity processes in astrocytes. We have demonstrated a link between the HA and TLR-mediated responses in astrocytes. This link is rather complex and includes mechanisms that allow the modulation of pro- and anti-inflammatory cytokines and oxylipin synthesis with the involvement of the COX metabolic branch of arachidonic acids and the lipoxygenase branch of DHA metabolism. The precise molecular mechanisms of this link are a matter of further research, but there is no doubt that HA, in both LMW and HMW forms, plays an important role in the TLR signaling pathway in astrocytes, although this role is not simply of an anti-inflammatory type for HMW HA or of a pro-inflammatory type for LMW HA.

## 4. Materials and Methods

### 4.1. Reagents

Lipopolysaccharide (LPS) (Sigma-Aldrich, cat.no L2630 St. Louis, MO, USA), Poly I:C (PIC) (cat.no tlrl-pic, InvivoGen, San Diego, CA, USA) streptomycin–penicillin (cat.no A063), trypsin (cat.no P037), EDTA, fetal bovine serum (cat.no BS-110/500) were from PanEco (Moscow, Russia). Culture medium Dulbecco’s Modified Eagle Medium (DMEM) (cat.no 21885-025) (Gibco, Thermo Fisher Scientific, Waltham, MA, USA). High molecular weight hyaluronic acid (1.01–1.8 MDa, HMW) (sodium hyaluronate cat.no HA15M-1) and low molecular weight hyaluronic acid (41–65 KDa, LMW) (sodium hyaluronate cat.no HA40K-1), were from Lifecore biomedical (MN, USA). Antibodies against COX-2 (Cell Signaling Technology, D5H5, cat.no 12282, Danvers, MA, USA) and β-tubulin (Sigma Chemicals, Taufkirchen, Germany), secondary horseradish peroxidase conjugated antibodies (anti-rabbit, anti-mouse, and anti-goat) (SCBT and CST), western blotting substrate ECL (Thermo Fisher Scientific, cat.no 32209, Waltham, MA, USA), and ELISA kits for TNFα (cat.no. KRC3012) and IL-10 (cat.no. BMS629) (InvivoGen, San Diego, CA, USA) were also used. The oxylipins standards were as follows: Tetranor-PGEM-d6 (cat.no. 314840), 6-keto PGF1α-d4 (cat.no. 315210), TXB2-d4 (cat.no. 319030), PGF2α-d4 (cat.no. 316010), PGE2-d4 (cat.no. 314010), PGD2-d4 (cat.no. 312010), Leukotriene (LT) C4-d5 (cat.no. 10006198), LTB4-d4 (cat.no. 320110), 5(S)-HETE-d8 (cat.no. 334230), 12(S)-HETE-d8 (cat.no. 334570), 15(S)-HETE-d8 (cat.no. 334720), PAF C16-d4 (cat.no. 10010229), Oleoyl Ethanolamide-d4 (cat.no. 9000552), PGA2-d4 (cat.no. 310210)(Cayman Chemical, Ann Arbor, MI, USA). Oasis^®^ PRIME HLB cartridge (60 mg, 3cc, cat.no. 186008056) were obtained from Waters, Eschborn, Germany.

### 4.2. Primary Cell Culture

The cells were obtained from one- or two-day old pups of Wistar rats. All of the experimental procedures were performed according to the guidelines in the European Convention for the Protection of Vertebrate Animals used for Experimental and Other Scientific Purposes, and were approved by the Bioethics Committee (Protocol 2/13 from 8 April 2013) of The Department of Biology at the Moscow State University. The cultures of primary rat astrocytes were obtained from newborn rats of both sexes, as previously reported [28]. In brief, the brains from decapitated pups were rinsed with ice-cold Puck’s solution (137.0 mM NaCl, 5.4 mM KCl, 0.44 mM KH_2_PO_4_, 0.3 mM Na_2_HPO_4_, and 5.5 mM glucose, pH 7.4) and triturated against nylon meshes with the pores of 250 and 136 μm, in a consecutive order. The dissociated cells were plated into 75 cm^2^ culture flasks at a density of 6 × 10^5^ cells per mL. The cells were subsequently cultured in DMEM (1 g/L D-glucose, 10% bovine fetal serum [FBS], 50 units/mL streptomycin, 50 μg/mL penicillin) at 37 °C, with 10% CO_2_. After five days of cultivation in DMEM, the culture medium was replaced with a fresh medium and the flasks were placed on a shaker at 200 rpm for 4 h to dissociate the microglial cells. The microglia containing medium was discarded and the astrocytes-enriched cultures were further grown for the following four days, and the medium was replaced every two days. Subsequently, the cells were washed with phosphate buffered saline and detached from the plastic with trypsin–EGTA solution and plated into six-well plates, and were maintained for two days in DMEM. After this, the medium was replaced by the medium of the same composition, and the cells were used for the experiments. The stimulation with LPS was carried out in male and female astrocytes (100 ng/mL, 4 h). The LPS dosage was selected based on our previous studies [16,41]. In preliminary studies the MTT assay − (3-[4-dimethylthiazol-2-yl]-2,5-diphenyltetrazolium bromide) (Sigma Aldrich, St. Louis, MO, USA) assay was performed according to the manufacturer’s protocol for the HMW and LMW toxicity analysis (Figure S2). Briefly, the astrocytes after HA-treatment were seeded into 96-well plates. After being cultured at standard conditions, astrocytes were incubated with HMW (450 µg/mL) and LMW (450 µg/mL). After 48 h, 10 μL of MTT (5 mg/mL in PBS) solution was added to each well and then incubated for another 4 h. Then, the supernatant was discarded and 100 μL of DMSO was added to each well, shaking the plates for 10 min. The synergy H4 plate reader (BioTek, Winooski, VT, USA) was used to detect the absorbance at 570 nm.

### 4.3. Measurement of the Relative RNA Expression Level

Total mRNA was isolated using the GeneJET RNA Purification Kit (Thermo Scientific, Waltham, MA, USA). The concentration of RNA was measured using an Implen NanoPhotometer C. cDNA was generated according to the manufacturer’s instructions using the MMLV RT kit (Evrogen, Moscow, Russia) with oligo-(dT)-primers. Real-time PCR was performed using the 5x PCR-HS-SYBR mix (Evrogen, Moscow, Russia) and the DTlite 4 amplificator (DNATechnology, Moscow, Russia). The sequences of PCR primers used in this study were as follows: β-actin: forward 5′-TCATCACTATCGGCAATGAGCGGT-3′, reverse 5′ACAGCACTGTGTTGGCATAGAGGT3′; TNFα: forward 5′-CAAGGAGGAGAAGTTCCCAA-3′ reverse 5′-TGATCTGAGTGTGAGGGTCTG-3′; IL-10: forward 5′-CCCAGAAATCAAGGAGCATTTG-3′, reverse 5′-TCATTCTTCACCTGCTCCAC-3′; IL-6 forward 5′-CTGGTCTTCTGGAGTTCCGT-3′, reverse 5′-TGGTCTTGGTCCTTAGCCAC-3′, iNOS forward 5′-CCACAATAGTACAATACTACTTGG-3′, reverse 5′-ACGAGGTGTTCAGCGTGCTCCACG-3′ the annealing temperature was 57 °C. Expression of each gene was measured in 25 µL reactions using cDNA synthesized from 70 ng RNA per reaction well. The relative mRNA expression level was determined by the Δ*C*_T_ method. The β-actin gene was used as a constitutive gene for normalization. The level of normalized gene expression in control cells or in stimulated cells (specified directly in the text) was taken as one.

### 4.4. Western Blot Analysis

The astrocytes were lysed in a modified radio immunoprecipitation assay (RIPA) buffer (50 mM Tris, pH 7.4, 1% NP-40 Sigma Chemicals, 0.25% Na-deoxycholate, 150 mM NaCl, 1 mM EDTA, 1 mM Na_3_VO_4_, 1 mM NaF) and protease inhibitor cocktail (Roche Molecular Biochemicals, Mannheim, Germany). The protein concentration was determined by the standard Bradford assay. Samples containing 20 μg of protein in a conventional Laemmli buffer were loaded on each lane of a 10% sodium dodecyl sulfate-polyacrylamide gel and subjected to a standard SDS-PAGE. After electrophoresis, the proteins were transferred onto the nitrocellulose membrane with 0.2 μm pores. The membranes were blocked in a 10% Rotiblock (Roth, Nürnberg, Germany) solution for 1 h and subsequently subjected to a Phosphate-Buffered Saline with Tween 20 0.05%, with a respective primary antibody—anti-COX-2 (1:2000) at 4 °C overnight. Secondary species-specific antibodies (Dianova, Hamburg, Germany) were applied at the concentration of 1:10,000 for 1 h at room temperature. The conjugates were visualized using the SuperSignal™ West Femta Chemiluminescent Substrate (Thermo Scientific). For the β-tubulin analysis, the membranes were stripped at 21 °C for 20 min with the Restore Western Blot Stripping Buffer (Pierce, Bonn, Germany). The membranes were re-probed with an antibody against β-tubulin (1:10.000) from Sigma Chemicals, and secondary anti-mouse IgG (Dianova, Hamburg, Germany), to control for protein loading. The protein bands were visualized by the SuperSignal™ West Pico Chemiluminescent Substrate (Thermo Scientific). Densitometry was carried out on four different experiments. The band intensity was measured using a GS-800 calibrated densitometer signal and Quantity One software (Bio-Rad, Hercules, CA, USA), and normalized to the intensity of the respective bands obtained for β-tubulin.

### 4.5. Immunofluorescence Analysis

The astrocytes were plated onto glass-bottom Petri dishes at the quantity of 10^5^ cells/glass and allowed to attach for 12 h. After the media change, the cells were left for an additional 24 h and used in the experiments, as described elsewhere. The slides with cells fixed in 4% paraformaldehyde buffered with PBS and were treated with Triton X-100 containing the buffer, and were blocked with FBS and subsequently incubated overnight with primary antibodies against GFAP (1:2000). The Alexa secondary antibodies, from goat, were used at the following dilutions: Alexa 488 anti-rabbit 1:1000. The negative control involved incubating astrocytes with only secondary antibodies. No significant staining was observed in the negative controls. The images are representative of three independent experiments. The images were obtained with an Axiovert.A1 HBO50 (Zeiss, Göttingen, Germany), equipped with the microscopy software ZEN 2.3. Images were processed using the ImageJ (1.51s) software (National Institute of Health, Bethesda, Maryland, USA).

### 4.6. UPLC-MS/MS Conditions and Sample Preparation

After the experiments, the supernatant was collected and stored at −70 °C for further analysis. The cell-free culture media were taken for the solid-phase lipid extraction (Oasis^®^ PRIME HLB cartridge (60 mg, 3cc)). A half ml of the prepared sample was loaded onto the column and washed with 1 mL 0.1% formic acid and 1 mL 15% methanol. The cartridges were then eluted with 500 μL of methanol and 500 μL of Acetonitrile, and then the solvent was evaporated under gentle stream of nitrogen. The lipid mediators were analyzed by the 8040 series UPLC-MS/MS (Shimadzu, Kyoto, Japan), with all of the specifications set as previously reported [25]. The quantification and qualification were accomplished in the multiple-reaction monitoring mode, and the MS was operated at a unit mass resolution for both the precursor and product ions. The Lipid Mediator Version 2 software package was used to operate the mass spectrometer (Shimadzu, Japan). The mediators were separated based on their chemical properties in UPLC, then, we monitored their ion fragments by a collision-induced dissociation in conjunction with the electrospray ionization-MS/MS. Lipids were identified according to accurate *m*/*z*, retention time, relative retention time of species in the same class, and the spectra of MS/MS. For the quantitative analysis of oxylipins, all of the samples were examined by LC-MS/MS to measure the peak areas of the detected species. In order to compensate for the fluctuations in MS intensities during different runs, the peak areas of each individual lipid species were corrected by deuterated internal standards. The concentration of lipids was normalized to the total protein and was expressed as pg/mg. The total protein was determined by the Bradford assay.

### 4.7. Determination of TNFα and IL-10 by Enzyme-Linked Immunoassay

After the experiments, supernatants were collected and stored at −70 °C for further analysis. The levels of the released TNFα and IL-10 were determined using an enzyme-linked immunoassay commercial kits and Synergy H4 plate reader (BioTek, Winooski, VT, USA), following the manufacturer’s instructions.

### 4.8. Experimental Data Analysis and Statistics

The data are expressed as mean ± SEM. The normality of data sets was assessed using the Shapiro-Wilk test. The data were subjected to a one-way ANOVA, followed by Bonferroni’s post hoc test, in order to determine the statistical significance. *p* < 0.05 was considered statistically significant. All of the experiments were repeated at least three times.

## Supplementary Materials

Supplementary materials can be found at https://www.mdpi.com/1422-0067/20/16/3894/s1.

## Abbreviations

HA	hyaluronic acid
HMW	high molecular weight
LMW	low molecular weight
TLR	toll-like receptor
LPS	lipopolysaccharide
TNFα	tumor necrosis factor alpha
COX-2	cyclooxygenase-2
DHA	docosahexaenoic acid
AA	arachidonic acid
IL-10	interleukin-10
iNOS	inducible nitric oxide synthase
IL-6	interleukin 6
