# Supplementary material for: High and Low Molecular Weight Hyaluronic Acid Differentially Influences Oxylipins Synthesis in Course of Neuroinflammation
Source: Int J Mol Sci. 2019 Aug 9;20(16):3894. doi: 10.3390/ijms20163894 (PMC6719050; doi:10.3390/ijms20163894)
Supplement: Supplementary file 1 [file ijms-20-03894-s001.pdf]

High and low molecular weight hyaluronic acid differentially influences oxylipins synthesis in course of neuroinflammation

Supplementary Information

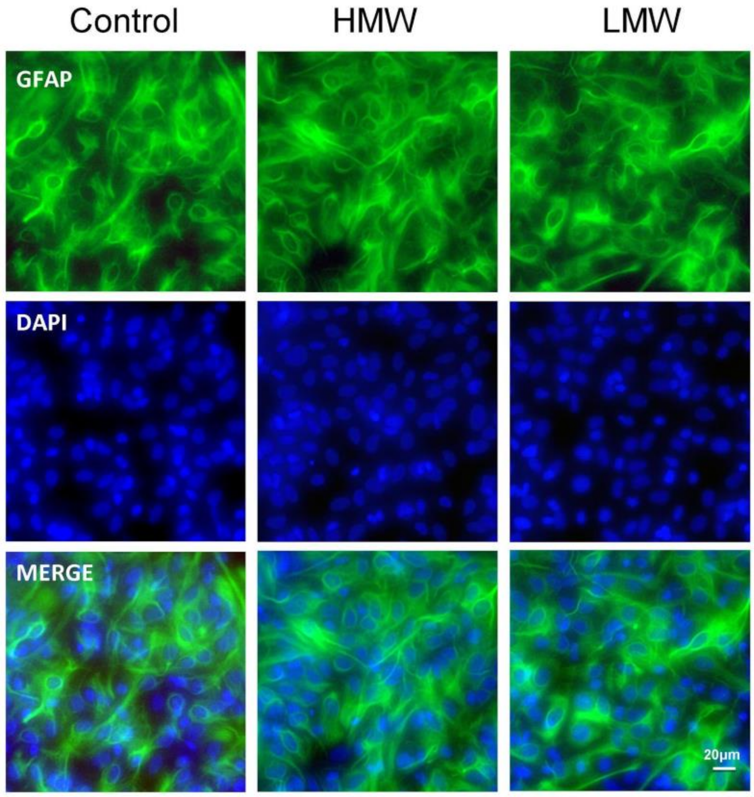

Figure S1. Representative immunofluorescence images showing astrocytes cell culture morphology treated with high (HMW) and low (LMW) molecular weight HA for 48h. 40X immunofluorescence microscopy using Rabbit Anti-GFAP (Green) monoclonal antibody visualized with Anti-Rabbit Alexa Fluor® 488 secondary antibody and DAPI stain for nucleus identification. Scale Bar = 20 μm.

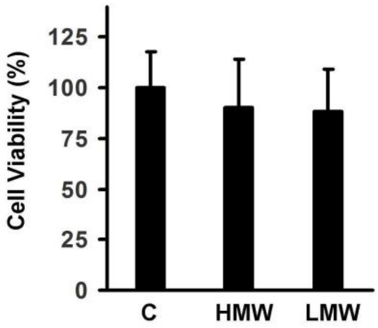

Figure S2. Cell viability was determined by MTT assay in cells treated for 48 h. Astrocytes were incubated without HA (control, C) with HMW (450 μg/ml), or with LMW (450 μg/ml) Data are mean ± SD of four independent experiments. *$p < 0.05$ vs. control.
